# Before writing that script: use of antipsychotic medication in patients with dementia in a CMHTOA

**DOI:** 10.1192/bjo.2021.289

**Published:** 2021-06-18

**Authors:** Adaora Obiekezie, Roohi Afshan, Jack Healy

**Affiliations:** 1 Surrey and Borders Partnership NHS Foundation Trust; 2 Surrey County Council

## Abstract

**Aims:**

A 2009 independent review commissioned by the UK government to review the use of antipsychotic medication in patients diagnosed with dementia produced the Time for Action report, often referred to as the Banerjee Report. It highlighted the common practise of using antipsychotics in the treatment of Behavioural and Psychological Symptoms of Dementia (BPSD) and the clinical issues this could raise especially when these medications were not being regularly reviewed. The audti was therefore carried out to determine whether patinets with BPSD in a Community Mental Health Team for Older adults (CMHTOA) in Mid Surrey, who had been diagnosed with BPSD, were being adequately assessed and managed in line with the current guidelines.

**Method:**

Patients with a diagnosis of dementia open to one of three teams in the CMHTOA during the months of October and November 2019 were identified, those being prescribed antipsychotic medication were selected and data from their electronic records collected and analysed to determine if clinicians: a) identified and documented the target behaviours, b) carried out a structured assessment using an ABC chart before commencing medication, c) reviewed the antipsychotic medication 6 weeks after it was commenced.

**Result:**

Of the 87 patients with a diagnosis of Dementia from October to November 2019, 18 were on antipsychotic medication. 100% of these had target behaviours identified and clearly documented, a sixth had a structured assessment prior to starting medication and 61% had been reviewed after the first 6 weeks of starting antipsychotics.

**Conclusion:**

The findings showed that a good proprotion of patients did not have the required structured assessment before commencement of treatment and that more needed to be done by way of improving regular reviews after antipsychotic treatment is commenced.

